# Supplementary material for: Single-cell transcriptomics and cell-specific proteomics reveals molecular signatures of sleep
Source: Commun Biol. 2022 Aug 19;5:846. doi: 10.1038/s42003-022-03800-3 (PMC9391396; doi:10.1038/s42003-022-03800-3)
Supplement: Supplementary file 2 — Supplementary Information [file 42003_2022_3800_MOESM2_ESM.pdf]

Supplementary Information

Single-cell transcriptomics and cell-specific proteomics reveals molecular signatures of sleep

Pawan K. Jha, Utham K. Valekunja, Sandipan Ray, Mathieu Nollet & Akhilesh B. Reddy

Supplementary Information (available in this document)

Supplementary Figures 1 – 8
Supplementary Table 1-10
Supplementary References

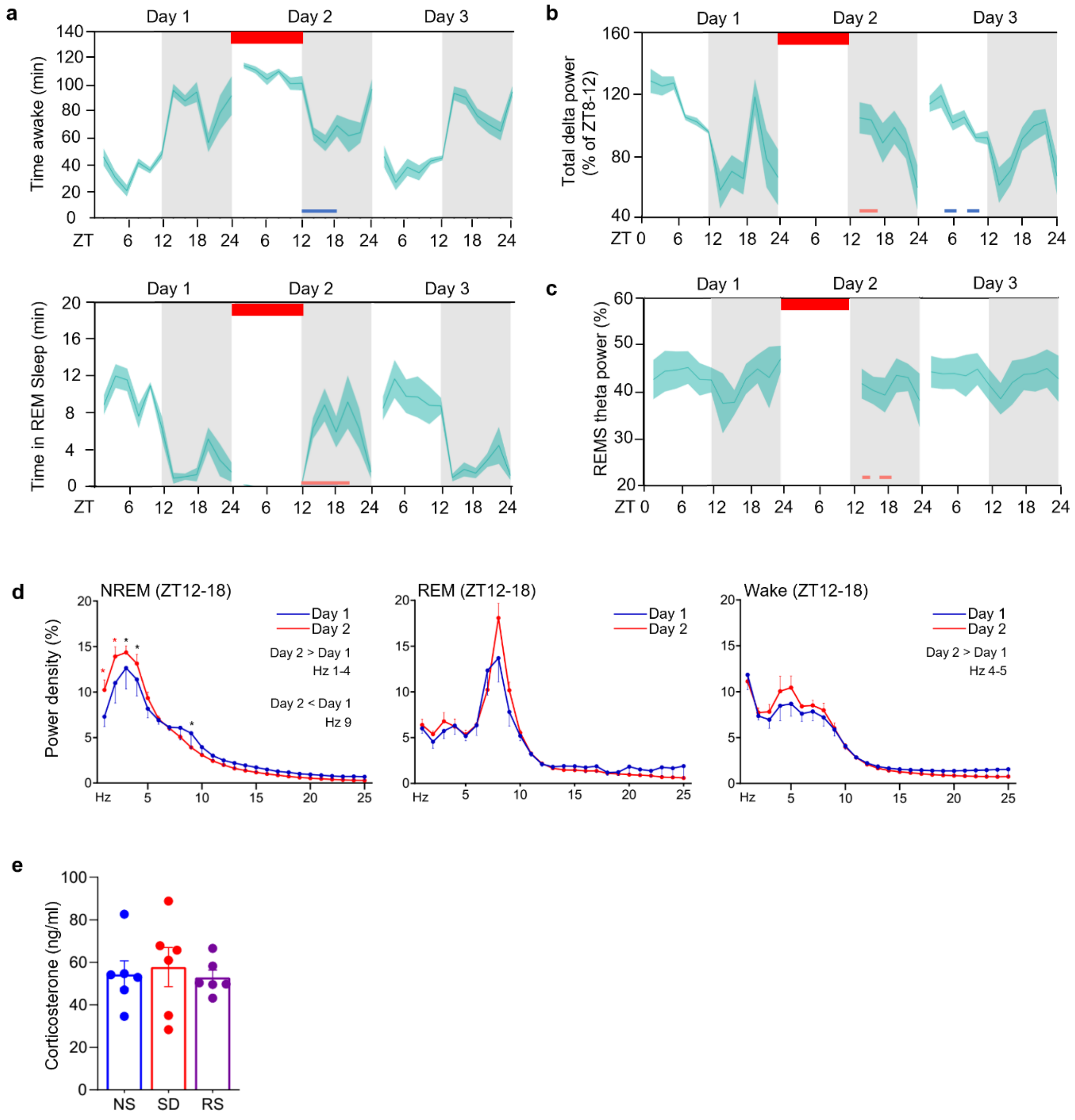

Supplementary Fig. 1: Sleep phenotyping and plasma corticosterone measurements during sleep treatments

a, **b**, **c** Ribbon plots showing the time course of 2 hourly binned values of (**a**) wake and REM sleep states, (**b**) total delta (δ)-power percentage of ZT8-12 from Day 1 and (**c**) REM theta (θ)-power. Horizontal red bar represents the timeline of sleep deprivation (Day 2, ZT 0-12; 12 h duration). Pink and blue bars under the plots denote significant increase and decrease, respectively, compared to Day 1 ($P < 0.05$). (**d**) Relative EEG power spectra from ZT 12-18 on Day 1 versus Day 2. (**e**) Bar plot showing unaltered plasma corticosterone concentration following sleep treatments. Data are mean±s.e.m.. n = 6 biological replicates. Repeated-measures two-way ANOVA (a-d) and one-way ANOVA (**e**) followed by post-hoc Fisher's Least Significant Difference (LSD) test. *(black), P < 0.01; *(red), P < 0.001. For sample sizes and statistics, see Supplementary Data 1. NS: normal sleep, SD: sleep deprived, RS: recovery sleep.

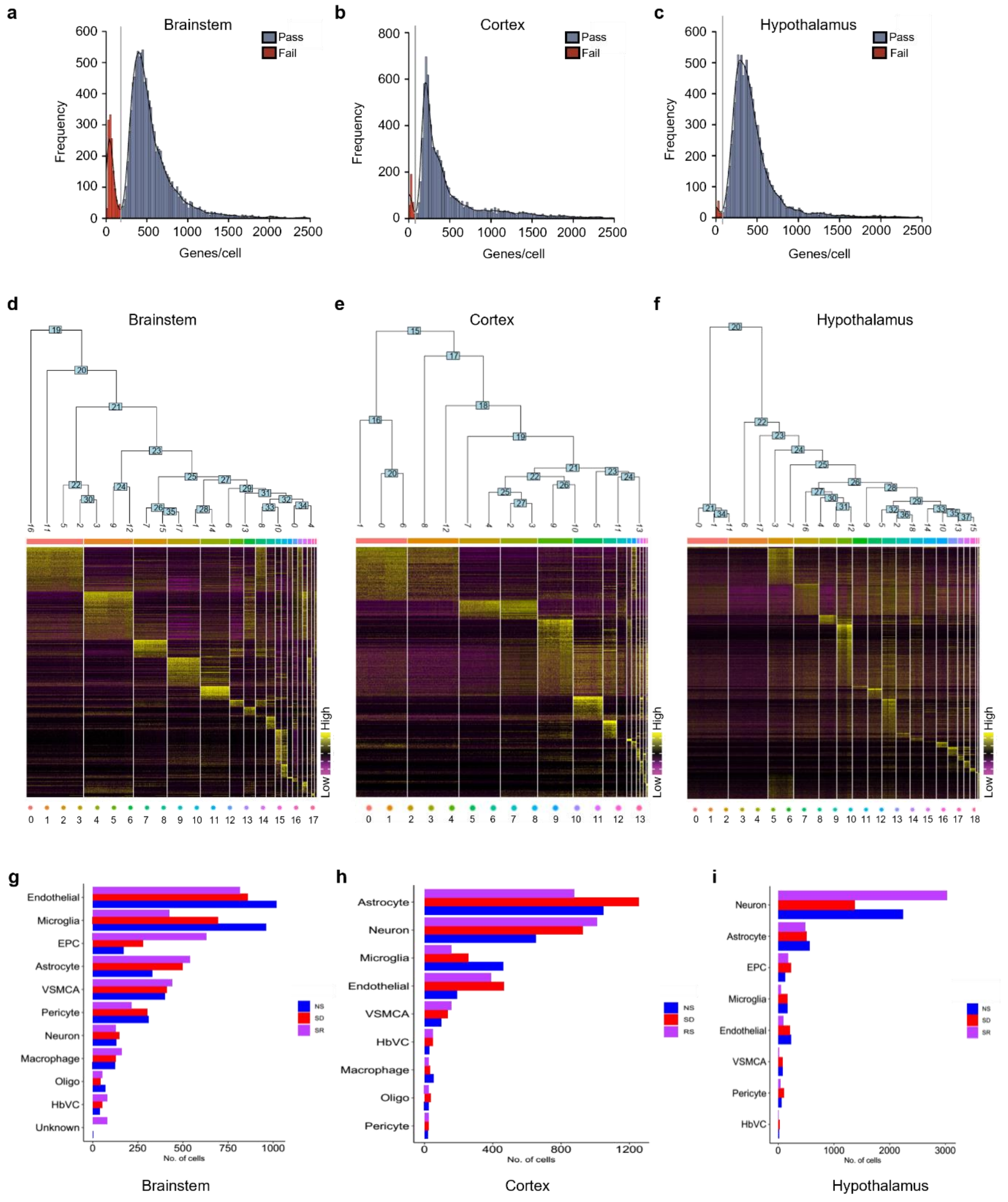

Supplementary Fig. 2: Quality control and identification of cell clusters based on specific expression patterns in the brainstem, cortex, and hypothalamus

a, **b**, **c** Distribution of genes per cell in (**a**) brainstem, (**b**) cortex, and (**c**) hypothalamus. **d**, **e**, **f** Heatmap and dendrogram showing distinct expression clusters for gene markers in (**d**) brainstem, (**e**) cortex, and (**f**) hypothalamus. **g**, **h**, **i** Bar chart showing the number of cell types identified across Normal sleep (NS), Sleep deprived (SD), and Recovery Sleep (RS) in (**g**) brainstem, (**h**) cortex, and (**i**) hypothalamus.

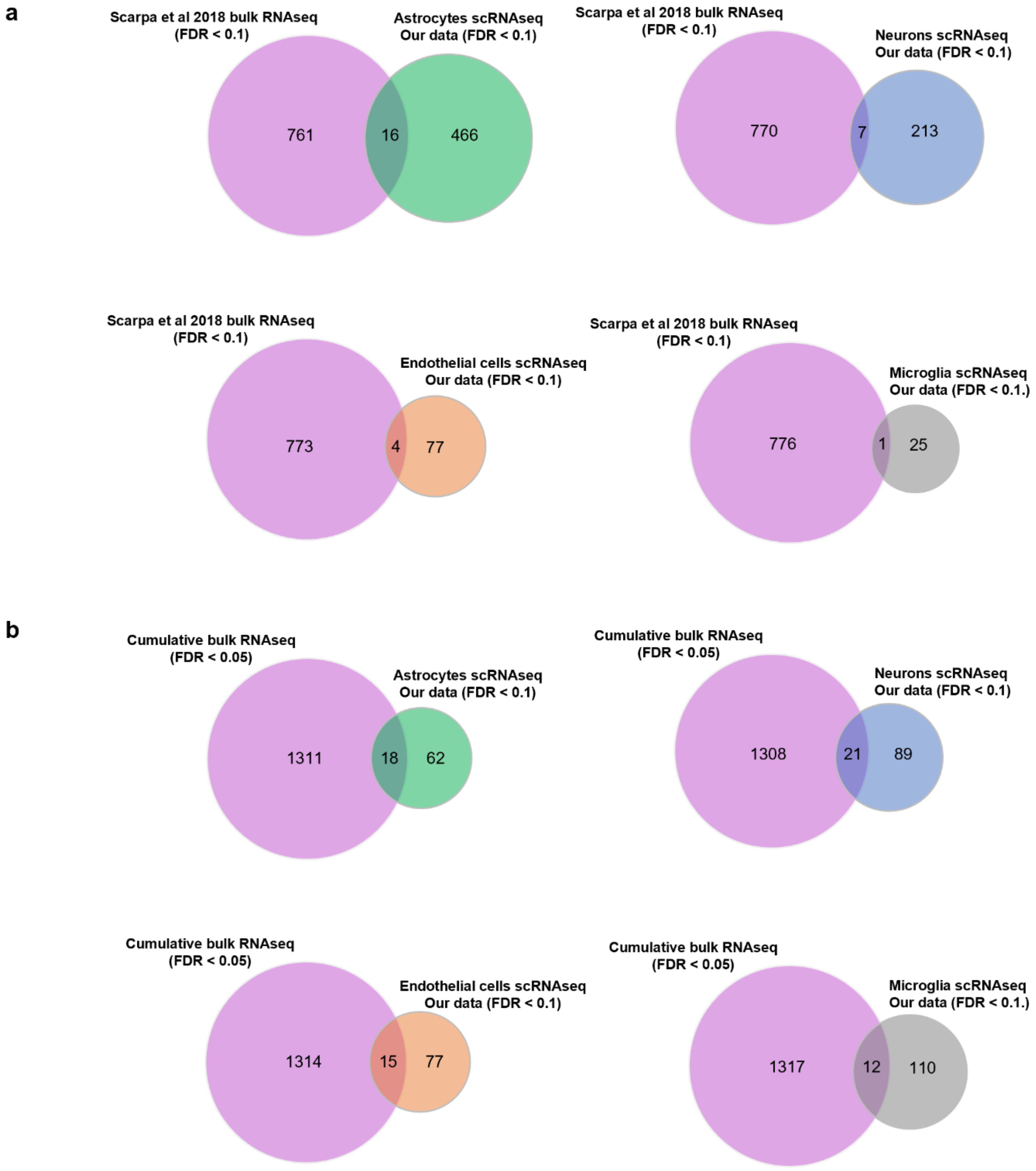

Supplementary Fig. 3: Cell-type specificity of previously published sleep related transcriptional studies compared to scRNAseq results

a, **b** Venn diagrams of sleep related transcriptional alterations at cumulative bulk cell and single-cell (astrocytes, neurons, endothelial cells, and microglia) in (**a**) cortex, and (**b**) hypothalamus. Cumulative bulk cell transcription studies were Diessler et al., 2018; Gerstner et al., 2016; Scarpa et al., 2018. See also Supplementary Table 4.

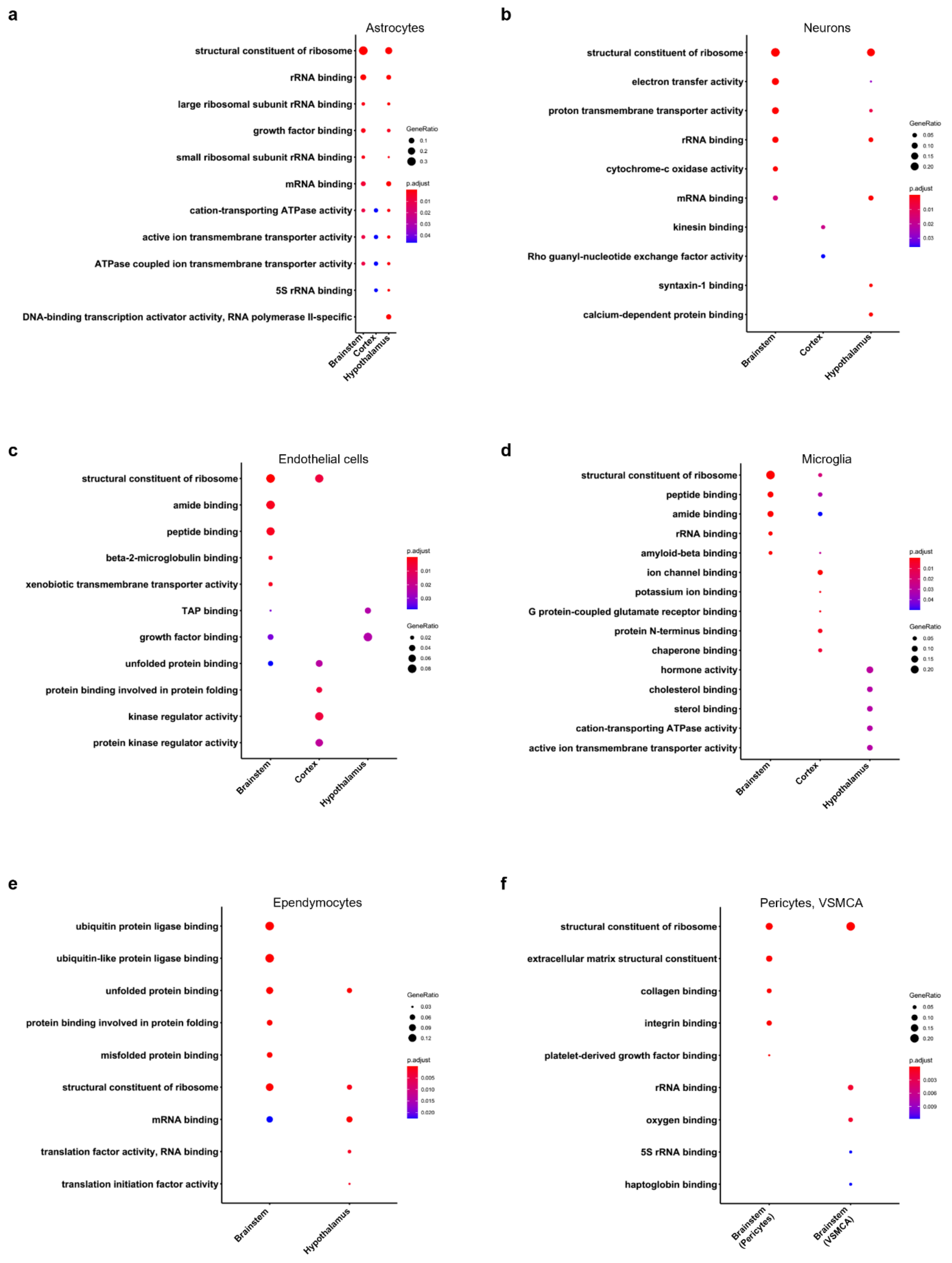

Supplementary Fig. 4: Molecular functions of differentially expressed genes in the cells from brainstem, cortex, and hypothalamus across sleep treatments

a, **b**, **c**, **d**, **e**, **f** Dot plots showing the comparison of significantly enriched (Benjamini-Hochberg adjusted *P*-value < 0.01) gene ontology (GO) terms of molecular functions in (**a**) astrocyte, (**b**) neuron, (**c**) endothelial cells, (**d**) microglia, (**e**) ependymocytes, (**f**) pericytes and VSMCA of

all three brain regions. Circle size is proportional to gene ratio and level of significance (Benjamini-Hochberg adjusted *P*-value) is colour coded.

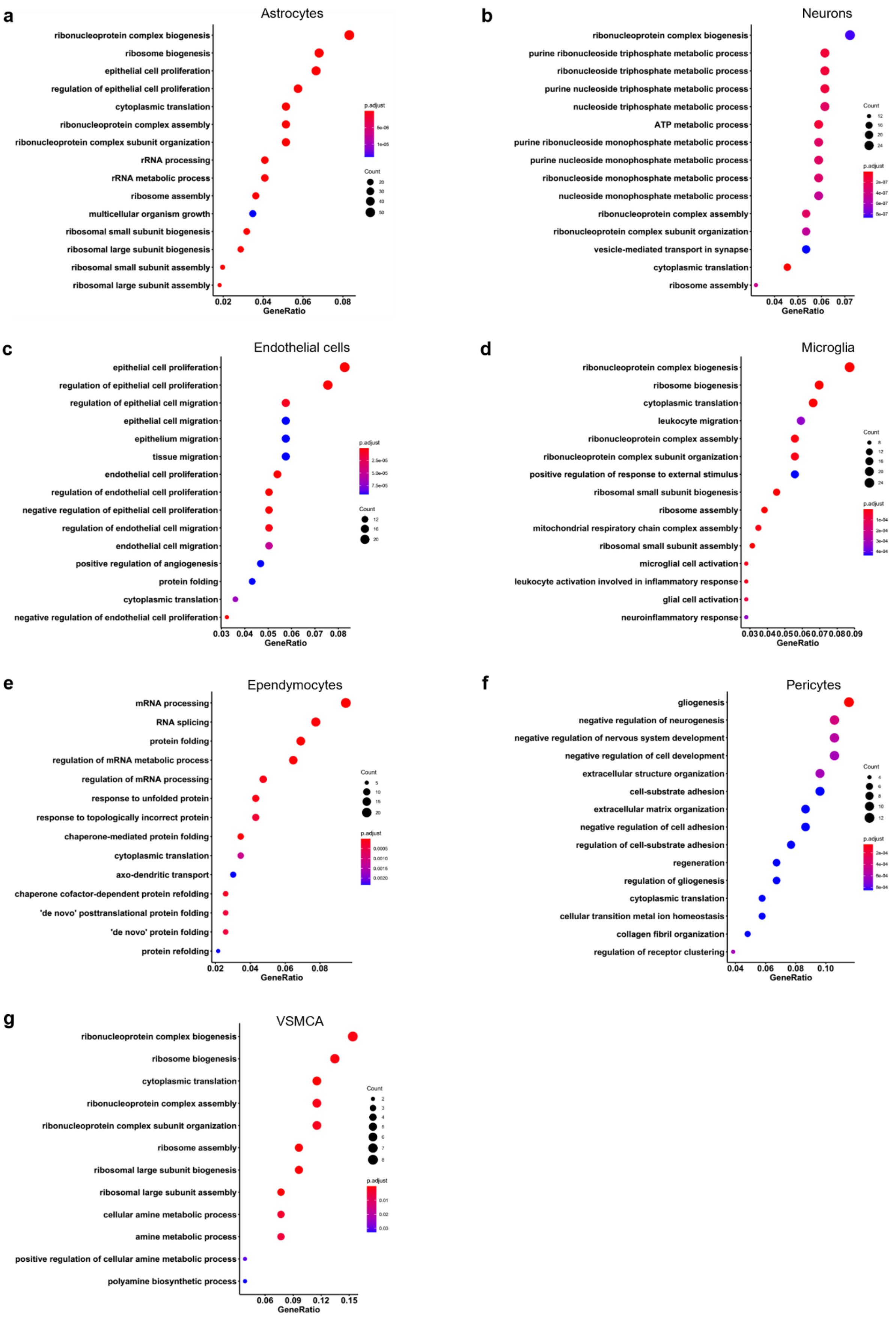
a
Astrocytes
ribonucleoprotein complex biogenesis
ribosome biogenesis
epithelial cell proliferation
regulation of epithelial cell proliferation
cytoplasmic translation
ribonucleoprotein complex assembly
ribonucleoprotein complex subunit organization
rRNA processing
rRNA metabolic process
ribosome assembly
multicellular organism growth
ribosomal small subunit biogenesis
ribosomal large subunit biogenesis
ribosomal small subunit assembly
ribosomal large subunit assembly
0.02 0.04 0.06 0.08
GeneRatio
p.adjust
Count
b
Neurons
ribonucleoprotein complex biogenesis
purine ribonucleoside triphosphate metabolic process
ribonucleoside triphosphate metabolic process
purine nucleoside triphosphate metabolic process
nucleoside triphosphate metabolic process
ATP metabolic process
purine ribonucleoside monophosphate metabolic process
purine nucleoside monophosphate metabolic process
ribonucleoside monophosphate metabolic process
nucleoside monophosphate metabolic process
ribonucleoprotein complex assembly
ribonucleoprotein complex subunit organization
vesicle-mediated transport in synapse
cytoplasmic translation
ribosome assembly
0.04 0.05 0.06 0.07
GeneRatio
Count
p.adjust
c
Endothelial cells
epithelial cell proliferation
regulation of epithelial cell proliferation
regulation of epithelial cell migration
epithelial cell migration
epithelium migration
tissue migration
endothelial cell proliferation
regulation of endothelial cell proliferation
negative regulation of epithelial cell proliferation
regulation of endothelial cell migration
endothelial cell migration
positive regulation of angiogenesis
protein folding
cytoplasmic translation
negative regulation of endothelial cell proliferation
0.03 0.04 0.05 0.06 0.07 0.08
GeneRatio
p.adjust
Count
d
Microglia
ribonucleoprotein complex biogenesis
ribosome biogenesis
cytoplasmic translation
leukocyte migration
ribonucleoprotein complex assembly
ribonucleoprotein complex subunit organization
positive regulation of response to external stimulus
ribosomal small subunit biogenesis
ribosome assembly
mitochondrial respiratory chain complex assembly
ribosomal small subunit assembly
microglial cell activation
leukocyte activation involved in inflammatory response
glial cell activation
neuroinflammatory response
0.03 0.04 0.05 0.06 0.07 0.08 0.09
GeneRatio
Count
p.adjust
e
Ependymocytes
mRNA processing
RNA splicing
protein folding
regulation of mRNA metabolic process
regulation of mRNA processing
response to unfolded protein
response to topologically incorrect protein
chaperone-mediated protein folding
cytoplasmic translation
axo-dendritic transport
chaperone cofactor-dependent protein refolding
'de novo' posttranslational protein folding
'de novo' protein folding
protein refolding
0.02 0.04 0.06 0.08
GeneRatio
Count
p.adjust
f
Pericytes
gliogenesis
negative regulation of neurogenesis
negative regulation of nervous system development
negative regulation of cell development
extracellular structure organization
cell-substrate adhesion
extracellular matrix organization
negative regulation of cell adhesion
regulation of cell-substrate adhesion
regeneration
regulation of gliogenesis
cytoplasmic translation
cellular transition metal ion homeostasis
collagen fibril organization
regulation of receptor clustering
0.04 0.06 0.08 0.10
GeneRatio
Count
p.adjust
g
VSMCA
ribonucleoprotein complex biogenesis
ribosome biogenesis
cytoplasmic translation
ribonucleoprotein complex assembly
ribonucleoprotein complex subunit organization
ribosome assembly
ribosomal large subunit biogenesis
ribosomal large subunit assembly
cellular amine metabolic process
amine metabolic process
positive regulation of cellular amine metabolic process
polyamine biosynthetic process
0.06 0.09 0.12 0.15
GeneRatio
Count
p.adjust

Supplementary Fig. 5: Biological functions of differentially expressed genes in the cells from brainstem, cortex, and hypothalamus across sleep treatments

a, **b**, **c**, **d**, **e**, **f**, **g** Dot plots showing significantly enriched (Benjamini-Hochberg adjusted *P*-value < 0.01) gene ontology (GO) terms of biological processes in (**a**) astrocyte, (**b**) neuron, (**c**) endothelial cells, (**d**) microglia, (**e**) ependymocytes, (**f**) pericytes and (**g**) VSMCA. Circle size is proportional to genes count and level of significance (Benjamini-Hochberg adjusted *P*-value) is colour coded.

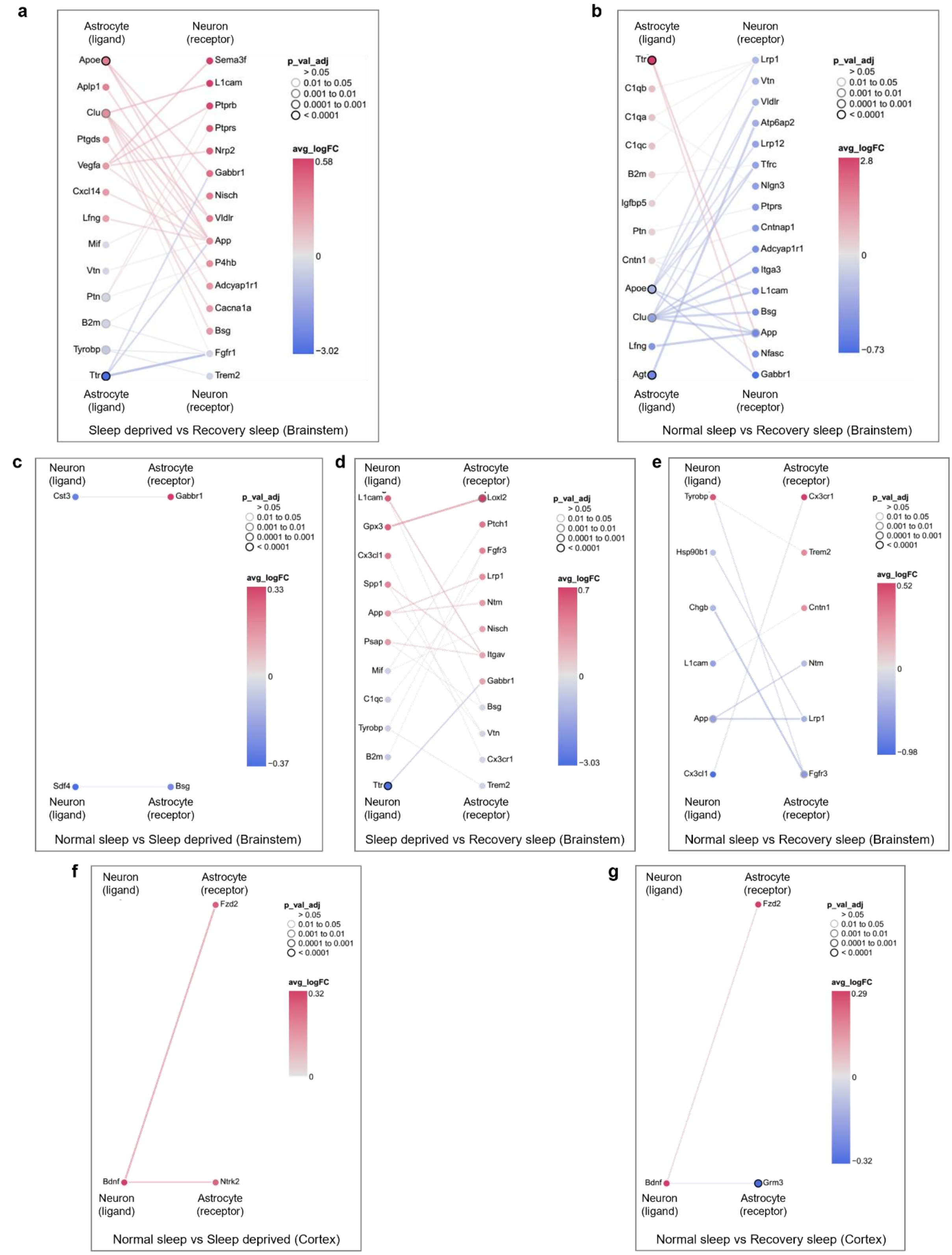

Supplementary Fig. 6: Sleep need modulates cell-cell communications networks in the brainstem and cortex

a, **b**, **c**, **d**, **e** Interactions between differentially expressed ligands of astrocytes and receptors of neurons for (**a**) SD versus RS, (**b**) NS versus RS and, ligands of neurons and receptors of

astrocytes for (**c**) NS versus SD, (**d**) SD versus RS, and (**e**) NS versus RS comparisons in the brainstem. **f**, **g** Interactions between differentially expressed ligands of neurons and receptors of astrocytes for (**f**) NS versus SD and (**g**) NS versus RS comparisons in the cortex. Nodes represent ligands or receptors in astrocytes and neurons, respectively. Node outline thickness indicates the level of significance (Benjamini-Hochberg adjusted *P*-value). Colour of nodes and edges represent the magnitude of alteration in expression (log fold-change, logFC) (see Methods). NS: normal sleep, SD: sleep deprived, RS: recovery sleep.

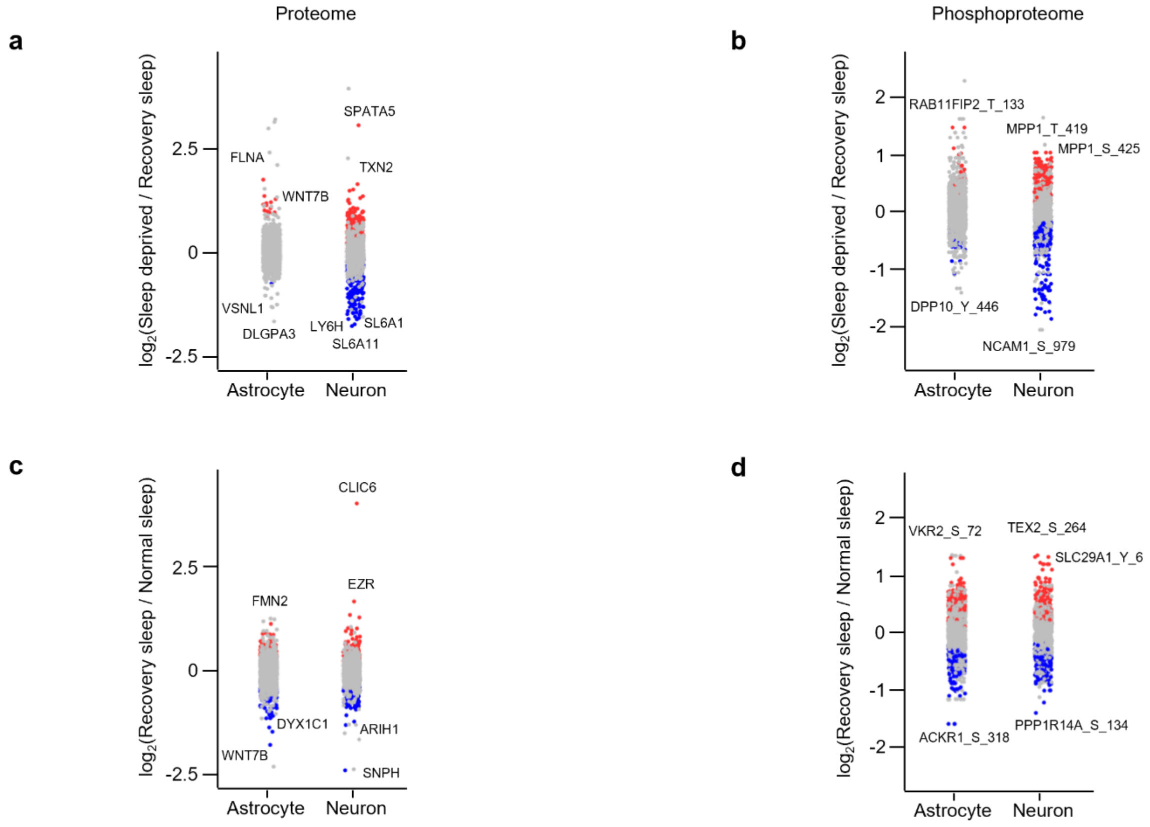

Supplementary Fig. 7: Translational and post-translational profiles of astrocytes and neurons from cortex across sleep-wake states

a, **b**, **c**, **d** Strip chart showing changes in (**a**) global and (**b**) phosphorylated proteins for Sleep Deprived (SD)/Recovery Sleep (RS), whereas (**c**) and (**d**) show changes in global and phosphorylated proteins, respectively for Recovery Sleep (RS)/Normal Sleep (NS) comparisons. Unpaired *t*-tests followed by false discovery rate (FDR) analysis were used to compare groups. Significantly upregulated and downregulated protein expression is colour coded with red and blue, respectively (Benjamini-Hochberg adjusted *P*-value < 0.1). Proteins in grey are not significantly changed following sleep treatments.

a

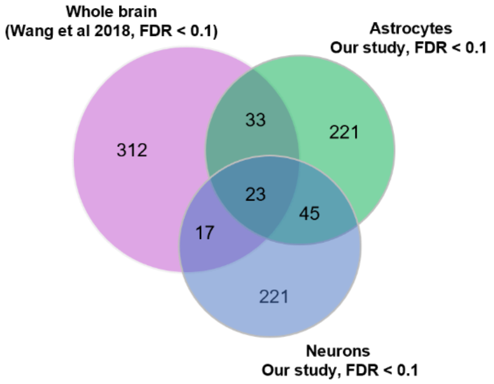

Supplementary Fig. 8: Cell-type specificity of previously published sleep related translational study compared to scRNAseq results

a Venn diagram of sleep driven alterations in protein phosphorylation in the whole brain and, astrocytes and neurons. Whole brain protein phosphorylation data from Wang et al., 2018. See also Supplementary Table 8.

**Cell type**	**Markers**
Astrocyte	*Aldoc*
Ependymocytes	*Tmem212, Rarres2*
Hemoglobin-expressing vascular cells	*Alas2*
Macrophage	*Lyz2*
Microglia	*Hexb, Cx3cr1*
Neuron Glutamatergic	*Snap25, Slc17a6, Slc17a7*
Neuron Other	*Snap25, Chga*
Neuron GABAergic	*Snap25, Gad2, Dlk1*
Neuron Inhibitory	*Snap25, Nrxn3*
Oligodendrocyte	*Cldn11*
Pericyte	*Vtn*
Vascular smooth muscle cells, arterial (VSMCA)	*Acta2*

Supplementary Table 1:

Summary of cell type classifications in single-cell transcriptomics.

**Brain area**	**Cell type**	**Significantly altered genes after sleep treatments**
Brainstem	Astrocyte	141
	Endothelial	169
	EPC	83
	Microglia	171
	Neuron	84
	Pericyte	124
	VSMCA	66
Cortex	Astrocyte	80
	Endothelial	92
	Microglia	122
	Neuron	110
Hypothalamus	Astrocyte	482
	Endothelial	58
	EPC	189
	Microglia	25
	Neuron	220
	Pericyte	3
	VSMCA	7

Supplementary Table 2:

Summary of differential gene expression within the cell types of brainstems, cortex, and hypothalamus.

Brain area	Cell Type	NS	SD	RS
Brainstem	Endothelial	28.60	25.19	22.88
	Microglia	27.05	20.38	11.94
	VSMCA	11.29	12.05	12.34
	Astrocyte	9.29	14.63	15.09
	Pericyte	8.73	8.83	6.04
	EPC	4.81	8.15	17.73
	Neuron	3.69	4.28	3.57
	Macrophage	3.52	3.75	4.47
	Oligo	1.94	1.23	1.49
	HbVC	1.07	1.50	2.25
	Unknown	0.03	0.00	2.19
Cortex	Astrocyte	40.60	39.49	39.49
	Neuron	25.33	29.15	29.15
	Microglia	17.83	8.10	8.10
	Endothelial	7.42	14.59	14.59
	VSMCA	3.85	4.29	4.29
	Macrophage	2.10	1.04	1.04
	HbVC	1.13	1.51	1.51
	Oligo	0.97	1.13	1.13
	Pericyte	0.78	0.69	0.69
Hypothalamus	Neuron	64.47	50.78	77.90
	Astrocyte	16.04	18.85	12.50
	Endothelial	6.47	7.69	2.31
	Microglia	4.92	6.10	1.26
	EPC	3.51	8.61	4.54
	VSMCA	2.36	3.10	0.28
	Pericyte	1.72	3.73	0.95
	HbVC	0.52	1.15	0.26

Supplementary Table 3:

Percentage of cell types in three sleep conditions from brainstem, cortex, and hypothalamus.

Brain area_Cell-type	Differentially altered genes in bulk RNAseq and scRNAseq	Upregulated genes	Downregulated genes
Cortex_Astrocyte	*Sdc4, Dbp, Timp4, Srsf2, Camk2n1, Htra1, Spry2, Mt2, Mfsd2a, Tmem100, Fam107a, Gjb6, Nim1k, Thrsp, Tsc22d3, Lrrc8a, Mat2a, Grm3*	*Sdc4, Timp4, Srsf2, Camk2n1, Htra1, Spry2, Mt2, Mfsd2a, Tmem100, Fam107a, Gjb6, Thrsp, Tsc22d3, Lrrc8a, Mat2a*	*Dbp, Nim1k, Grm3*
Cortex_Neuron	*Cbfa2t3, Sipa1l1, Chka, Llph, Sox1,1 Dpp10, Camk2n1, Frmd6, Bhlhe40, Spred2, Hsph1, Fscn1, Stmn4, Trio, Pja2, Mcf2l, Ttll7, Pdp1, Mast3, Homer1, Runx1t1*	*Cbfa2t3, Sipa1l1, Chka, Llph, Camk2n1, Frmd6, Bhlhe40, Spred2, Hsph1, Fscn1, Trio, Pdp1, Mast3, Homer1, Runx1t1*	*Sox11, Dpp10, Ttll7*
Cortex_Endothelial cell	*Itm2a, Pop5, Adgrl4, Srsf2, Camk2n1, Hnrnpf, Mat2b, Sgms1, Klf4, Klf13, Hspb1, Gkn3, Tsc22d3, 8430408G22Rik, Mat2a*	*Adgrl4, Srsf2, Camk2n1, Hnrnpf, Sgms1, Klf13, Tsc22d3, 8430408G22Rik, Mat2a*	*Itm2a, Gkn3*
Cortex_Microglia	*Rassf2, Sgk1, Krit1, Camk2n1, Spry2, Hexb, Mt2, Dclk1, Camk2a, Sdf2l1, Tsc22d3, Mat2a*	*Rassf2, Sgk1, Krit1, Camk2n1, Spry2, Mt2, Dclk1, Camk2a, Tsc22d3, Mat2a*	*Sdf2l1*
Hypothalamus_Astrocyte	*Ccnd2, H2-D1, Ncl, Slc7a10, Msl1, Kcnk2, Tsc22d1, Rps3, Socs3, Klf6, Errfi1, Slc6a6, Hspb1, Hnrnpa1, Wdr6, Ahi1*	*Socs3*	*Kcnk2, H2-D1, Ncl, Hnrnpa1, Wdr6, Msl1, Slc6a6*
Hypothalamus_Neuron	*Ccnd2, Cirbp, Arl6ip1, Serf2, Ncam1, Nrsn2, Per3*	*Cirbp, Serf2, Ncam1, Nrsn2*	*Ccnd2, Arl6ip1, Per3*
Hypothalamus_Endothelial cell	*Tnfaip2, Serf2, H2-D1, Fabp4*		*Tnfaip2, Serf2, H2-D1, Fabp4*
Hypothalamus_Microglia	*Ccnd2*		*Ccnd2*

Supplementary Table 4:

Cell-type specificity of previously reported sleep-related upregulated and downregulated genes in the cortex and hypothalamus.

Brain area	TF	co-expressed gene	GO terms	FDR
Brainstem	Sox2	*Ikzf2, Nr2f1*<br>*Sox2, Sox9*<br>*Trps1, Cd36*<br>*Gja1, Hey1*<br>*Kcnk2, Nkx6-1*<br>*Zbtb20*	Negative regulation of cellular macromolecule biosynthetic process	0.0023
	Nfe2l1	*Atp1b1, Grin1*<br>*Tnfrsf21, Scn1b*<br>*Rnf10*	Regulation of system process	0.0395
	Mafb	*Csf1r, Ralb*<br>*Rgs2, Pik3r1*<br>*Adrb2, Zeb2*	Regulation of kinase activity	0.00020
	Foxj1	*Cxxc5, Ezr*<br>*Hmgb1, Nfix*<br>*Txnip*	Negative regulation of RNA metabolic process	0.0253
	Zic1	*Lgf2, Lgfbp2*<br>*Nr2f2, Pdgfrb*	Cellular response to endogenous stimulus	0.0162
	Lef1	*Tshz1, Lef1*	Secondary palate development	0.0202
Cortex	Tef	*Susd4, Golga4*	Chromatin remodelling system [64]	0.0392
	Hlf	*Hlf, Csnk1d*	Biological rhythms	0.0345
	Cebpb	*Cebpb, Hes1*	Regulation of hemopoiesis	0.0039
	Sox9	*Fgfr1, Sox9*<br>*Hes5, Ppargc1a*	Regulation of protein phosphorylation	0.0019
Hypothalamus	Atf3	*Fos, Jun*<br>*Junb, Fosb*<br>*Dusp1, Actg1*	Response to calcium ion	1.04e-07
	Fosb	*Fos, Jun*<br>*Junb, Jund*<br>*Klf4, Lmna*<br>*Hspb1, Zfp36*<br>*Irs2, Ptfrf*<br>*Rorc*	Regulation of cell death	3.25e-08
	Mef2c	*Acta2, Tpm2*<br>*Mef2c, Chga*	Muscle system process	0.0037

Supplementary Table 5:

Gene annotation enrichment analysis results for uniquely expressed transcription factors and co-expressed target genes from cells in sleep-deprived mice.

Astrocyte (total expressed global proteins)	Astrocytes (differentially expressed global proteins across sleep treatments)
GOBP:0006091: generation of precursor metabolites and energy	GOBP:0050808: synapse organization
GOBP:0050808: synapse organization	GOBP:0061572: actin filament bundle organization
GOBP:0043254: regulation of protein complex assembly	GOBP:0043270: positive regulation of ion transport
GOBP:0015980: energy derivation by oxidation of organic compounds	GOBP:0050803: regulation of synapse structure or activity
GOBP:1990778: protein localization to cell periphery	GOBP:0032535: regulation of cellular component size
GOBP:0001505: regulation of neurotransmitter levels	GOBP:1902414: protein localization to cell junction
GOBP:0006887: exocytosis	GOBP:0034776: response to histamine
GOBP:0045333: cellular respiration	
GOBP:0046034: ATP metabolic process	
GOBP:0031346: positive regulation of cell projection organization	
GOBP:0032535: regulation of cellular component size	
GOBP:0098693: regulation of synaptic vesicle cycle	
GOBP:0032271: regulation of protein polymerization	
GOBP:0010256: endomembrane system organization	
GOBP:0007015: actin filament organization	
GOMF:0003779: actin binding	GOMF:0003779: actin binding
GOMF:0050662: coenzyme binding	GOMF:0043177: organic acid binding
GOMF:0051082: unfolded protein binding	GOMF:0022836: gated channel activity
GOMF:0032549: ribonucleoside binding	GOMF:0005216: ion channel activity
GOMF:0003924: GTPase activity	GOMF:0005244: voltage-gated ion channel activity
GOMF:0001882: nucleoside binding	GOMF:0022832: voltage-gated channel activity
GOMF:0003735: structural constituent of ribosome	GOMF:0015267: channel activity
GOMF:0005525: GTP binding	GOMF:0022803: passive transmembrane transporter activity
GOMF:0001883: purine nucleoside binding	
GOMF:0003729: mRNA binding	
GOMF:0005516: calmodulin binding	
GOMF:0035254: glutamate receptor binding	
GOMF:0042625: ATPase coupled ion transmembrane transporter activity	
GOMF:0000149: SNARE binding	
GOMF:0051087: chaperone binding	
GOCC:0043209: myelin sheath	GOCC:0032432: actin filament bundle
GOCC:0099572: postsynaptic specialization	GOCC:0015629: actin cytoskeleton
GOCC:0005743: mitochondrial inner membrane	GOCC:0001725: stress fiber
GOCC:0019866: organelle inner membrane	GOCC:0097517: contractile actin filament bundle
GOCC:0008021: synaptic vesicle	GOCC:0042641: actomyosin
GOCC:0070382: exocytic vesicle	GOCC:0005884: actin filament
GOCC:0030133: transport vesicle	GOCC:0099572: postsynaptic specialization
GOCC:0015629: actin cytoskeleton	
GOCC:0098798: mitochondrial protein complex	
GOCC:0031252: cell leading edge	
GOCC:0030659: cytoplasmic vesicle membrane	
GOCC:0012506: vesicle membrane	
GOCC:0005938: cell cortex	
GOCC:0030658: transport vesicle membrane	
GOCC:0022626: cytosolic ribosome	

Supplementary Table 6:

Functional annotations of total and differentially expressed global proteins in astrocytes across sleep treatments.

Neurons (total expressed global proteins)	Neurons (differentially expressed global proteins across sleep treatments)
GOBP:0006091: generation of precursor metabolites and energy	GOBP:0099504: synaptic vesicle cycle
GOBP:0043254: regulation of protein complex assembly	GOBP:0099003: vesicle-mediated transport in synapse
GOBP:0046034: ATP metabolic process	GOBP:0001505: regulation of neurotransmitter levels
GOBP:0008380: RNA splicing	GOBP:0006836: neurotransmitter transport
GOBP:0006897: endocytosis	GOBP:0036465: synaptic vesicle recycling
GOBP:0007015: actin filament organization	GOBP:0140029: exocytic process
GOBP:0006397: mRNA processing	GOBP:0006897: endocytosis
GOBP:0015980: energy derivation by oxidation of organic compounds	GOBP:0007269: neurotransmitter secretion
GOBP:0045333: cellular respiration	GOBP:0099643: signal release from synapse
GOBP:0032271: regulation of protein polymerization	GOBP:0097479: synaptic vesicle localization
GOBP:0001505: regulation of neurotransmitter levels	GOBP:0048488: synaptic vesicle endocytosis
GOBP:1902903: regulation of supramolecular fiber organization	GOBP:0140238: presynaptic endocytosis
GOBP:0051258: protein polymerization	GOBP:0006898: receptor-mediated endocytosis
GOBP:0099003: vesicle-mediated transport in synapse	GOBP:0016082: synaptic vesicle priming
GOBP:0099504: synaptic vesicle cycle	GOBP:0098693: regulation of synaptic vesicle cycle
GOMF:0003779: actin binding	GOMF:0019829: cation-transporting ATPase activity
GOMF:0051015: actin filament binding	GOMF:0042625: ATPase coupled ion transmembrane transporter activity
GOMF:0003729: mRNA binding	GOMF:0015662: ATPase activity, coupled to transmembrane movement of ions, phosphorylative mechanism
GOMF:0005200: structural constituent of cytoskeleton	GOMF:0000149: SNARE binding
GOMF:0050662: coenzyme binding	GOMF:0009678: hydrogen-translocating pyrophosphatase activity
GOMF:0031072: heat shock protein binding	GOMF:0022853: active ion transmembrane transporter activity
GOMF:0003735: structural constituent of ribosome	GOMF:0042626: ATPase activity, coupled to transmembrane movement of substances
GOMF:0019001: guanyl nucleotide binding	GOMF:0015399: primary active transmembrane transporter activity
GOMF:0032561: guanyl ribonucleotide binding	GOMF:0017075: syntaxin-1 binding
GOMF:0001882: nucleoside binding	GOMF:0051117: ATPase binding
GOMF:0032550: purine ribonucleoside binding	GOMF:0008022: protein C-terminus binding
GOMF:0032549: ribonucleoside binding	GOMF:0015077: monovalent inorganic cation transmembrane transporter activity
GOMF:0031625: ubiquitin protein ligase binding	GOMF:0044769: ATPase activity, coupled to transmembrane movement of ions, rotational mechanism
GOMF:0001883: purine nucleoside binding	GOMF:0046961: proton-transporting ATPase activity, rotational mechanism
GOMF:0044389: ubiquitin-like protein ligase binding	GOMF:0008553: proton-exporting ATPase activity, phosphorylative mechanism
GOCC:0043209: myelin sheath	GOCC:0043209: myelin sheath
GOCC:0015629: actin cytoskeleton	GOCC:0070382: exocytic vesicle
GOCC:0005743: mitochondrial inner membrane	GOCC:0008021: synaptic vesicle
GOCC:0019866: organelle inner membrane	GOCC:0030133: transport vesicle
GOCC:0014069: postsynaptic density	GOCC:0048786: presynaptic active zone
GOCC:0032279: asymmetric synapse	GOCC:0042734: presynaptic membrane
GOCC:0098984: neuron to neuron synapse	GOCC:0150034: distal axon
GOCC:0031252: cell leading edge	GOCC:0030672: synaptic vesicle membrane
GOCC:0099572: postsynaptic specialization	GOCC:0099501: exocytic vesicle membrane
GOCC:0005938: cell cortex	GOCC:0012506: vesicle membrane
GOCC:0070382: exocytic vesicle	GOCC:0030658: transport vesicle membrane
GOCC:0008021: synaptic vesicle	GOCC:0030659: cytoplasmic vesicle membrane
GOCC:0022626: cytosolic ribosome	GOCC:0098984: neuron to neuron synapse
GOCC:0098800: inner mitochondrial membrane protein complex	GOCC:0030426: growth cone
GOCC:0030133: transport vesicle	GOCC:0044306: neuron projection terminus

Supplementary Table 7:

Functional annotations of total and differentially expressed global proteins in neurons across sleep treatments.

Cell-type	Phosphorylated proteins
Astrocytes	*Ryr2, Map4k4, Stxbp5l, Camkv, Map7d1, Camsap1, Hid1, Pclo, Slc9a1, Rab11fip2, Rph3a, Ppp1r12a, Rap1gap, Mical3, Map1b, Bsn, Srgap3, Otud7a, Lysmd2, Mark4, Slc7a14, Htt, Pde4a, Ank2, Gtpbp1, Aak1, Amer2, Bclaf1, Csde1, Gm996, 2010300C02Rik, Ciapin1, Wdr7*
Neurons	*Pdha1, Ppp1r12b, Caskin1, Git2, Pi4ka, Map4, Clasp2, Tnks1bp1, Mark2, Oxr1, Gprin3, Pde4b, Plec, Raph1, Pkp4, Wnk2, Madd*

Supplementary Table 8:

Cell-type specificity to previously known sleep related phosphorylated proteins in cortex.

Astrocyte (total expressed phosphorylated proteins)	Astrocytes (differentially expressed phosphorylated proteins across sleep treatments)
GOBP:0050808: synapse organization	GOBP:0008361: regulation of cell size
GOBP:0007269: neurotransmitter secretion	GOBP:0032535: regulation of cellular component size
GOBP:1902903: regulation of supramolecular fiber organization	GOBP:0050808: synapse organization
GOBP:0043254: regulation of protein complex assembly	GOBP:0007269: neurotransmitter secretion
GOBP:0007015: actin filament organization	GOBP:0048588: developmental cell growth
GOBP:0006887: exocytosis	GOBP:0043254: regulation of protein complex assembly
GOBP:1901880: negative regulation of protein depolymerization	GOBP:0060560: developmental growth involved in morphogenesis
GOBP:1990778: protein localization to cell periphery	GOBP:0010769: regulation of cell morphogenesis involved in differentiation
GOBP:0070507: regulation of microtubule cytoskeleton organization	GOBP:0009187: cyclic nucleotide metabolic process
GOBP:0032271: regulation of protein polymerization	GOBP:0007015: actin filament organization
GOBP:0050803: regulation of synapse structure or activity	GOBP:0061387: regulation of extent of cell growth
GOBP:0043624: cellular protein complex disassembly	GOBP:1990778: protein localization to cell periphery
GOBP:0006897: endocytosis	GOBP:0032271: regulation of protein polymerization
GOBP:0051656: establishment of organelle localization	GOBP:0007163: establishment or maintenance of cell polarity
GOBP:0032535: regulation of cellular component size	GOBP:0043149: stress fiber assembly
GOMF:0051015: actin filament binding	GOMF:0005516: calmodulin binding
GOMF:0031267: small GTPase binding	GOMF:0003779: actin binding
GOMF:0005516: calmodulin binding	GOMF:0042301: phosphate ion binding
GOMF:0017016: Ras GTPase binding	
GOMF:0015631: tubulin binding	
GOMF:0019902: phosphatase binding	
GOMF:0005543: phospholipid binding	
GOMF:0003712: transcription coregulator activity	
GOMF:0005096: GTPase activator activity	
GOMF:0008017: microtubule binding	
GOMF:0004674: protein serine/threonine kinase activity	
GOMF:0019903: protein phosphatase binding	
GOMF:0060090: molecular adaptor activity	
GOMF:0008022: protein C-terminus binding	
GOMF:0044325: ion channel binding	
GOCC:0099572: postsynaptic specialization	GOCC:0099572: postsynaptic specialization
GOCC:0031252: cell leading edge	GOCC:0098589: membrane region
GOCC:0015629: actin cytoskeleton	GOCC:0008021: synaptic vesicle
GOCC:0005938: cell cortex	GOCC:0070382: exocytic vesicle
GOCC:0099568: cytoplasmic region	GOCC:0031252: cell leading edge
GOCC:0005874: microtubule	GOCC:0030426: growth cone
GOCC:0008021: synaptic vesicle	GOCC:0015629: actin cytoskeleton
GOCC:0070382: exocytic vesicle	GOCC:0030427: site of polarized growth
GOCC:0030427: site of polarized growth	GOCC:0030659: cytoplasmic vesicle membrane
GOCC:0030426: growth cone	GOCC:0019898: extrinsic component of membrane
GOCC:0030863: cortical cytoskeleton	GOCC:0030133: transport vesicle
GOCC:0031312: extrinsic component of organelle membrane	GOCC:0012506: vesicle membrane
GOCC:0030055: cell-substrate junction	GOCC:0099522: region of cytosol
GOCC:0099522: region of cytosol	GOCC:0030118: clathrin coat
GOCC:0043209: myelin sheath	GOCC:0099501: exocytic vesicle membrane

Supplementary Table 9:

Functional annotations of total and differentially expressed phosphorylated proteins in astrocytes across sleep treatments.

Neuron (total expressed phosphorylated proteins)	Neuron (differentially expressed phosphorylated proteins across sleep treatments)
GOBP:0032886: regulation of microtubule-based process	GOBP:0019932: second messenger-mediated signaling
GOBP:0007015: actin filament organization	GOBP:0097479: synaptic vesicle localization
GOBP:0007163: establishment or maintenance of cell polarity	GOBP:0019722: calcium-mediated signaling
GOBP:0034329: cell junction assembly	GOBP:0099504: synaptic vesicle cycle
GOBP:0010639: negative regulation of organelle organization	GOBP:0099003: vesicle-mediated transport in synapse
GOBP:0007264: small GTPase mediated signal transduction	GOBP:0043254: regulation of protein complex assembly
GOBP:0051656: establishment of organelle localization	GOBP:0098693: regulation of synaptic vesicle cycle
GOBP:0043244: regulation of protein complex disassembly	GOBP:0051656: establishment of organelle localization
GOBP:0097479: synaptic vesicle localization	GOBP:0006887: exocytosis
GOBP:0099003: vesicle-mediated transport in synapse	GOBP:0007269: neurotransmitter secretion
GOBP:0099504: synaptic vesicle cycle	GOBP:0099643: signal release from synapse
GOBP:0016358: dendrite development	GOBP:0050808: synapse organization
GOBP:0006397: mRNA processing	GOBP:0051258: protein polymerization
GOBP:0008088: axo-dendritic transport	GOBP:0070507: regulation of microtubule cytoskeleton organization
GOBP:0050808: synapse organization	GOBP:0007044: cell-substrate junction assembly
GOMF:0051015: actin filament binding	GOMF:0003779: actin binding
GOMF:0017016: Ras GTPase binding	
GOMF:0005516: calmodulin binding	
GOMF:0017048: Rho GTPase binding	
GOMF:0019903: protein phosphatase binding	
GOMF:0005088: Ras guanyl-nucleotide exchange factor activity	
GOMF:0015631: tubulin binding	
GOMF:0005543: phospholipid binding	
GOMF:0019902: phosphatase binding	
GOMF:0005089: Rho guanyl-nucleotide exchange factor activity	
GOMF:0008017: microtubule binding	
GOMF:0035091: phosphatidylinositol binding	
GOMF:0004112: cyclic-nucleotide phosphodiesterase activity	
GOMF:0003712: transcription coregulator activity	
GOMF:0004697: protein kinase C activity	
GOCC:0015629: actin cytoskeleton	GOCC:0014069: postsynaptic density
GOCC:0032279: asymmetric synapse	GOCC:0032279: asymmetric synapse
GOCC:0099572: postsynaptic specialization	GOCC:0098984: neuron to neuron synapse
GOCC:0098984: neuron to neuron synapse	GOCC:0099572: postsynaptic specialization
GOCC:0031252: cell leading edge	GOCC:0031252: cell leading edge
GOCC:0005911: cell-cell junction	GOCC:0015629: actin cytoskeleton
GOCC:0001725: stress fiber	GOCC:0005874: microtubule
GOCC:0005874: microtubule	GOCC:0150034: distal axon
GOCC:0005938: cell cortex	GOCC:0008021: synaptic vesicle
GOCC:0005925: focal adhesion	GOCC:0030133: transport vesicle
GOCC:0030863: cortical cytoskeleton	GOCC:0070382: exocytic vesicle
GOCC:0150034: distal axon	GOCC:0044306: neuron projection terminus
GOCC:0034399: nuclear periphery	GOCC:0098858: actin-based cell projection
GOCC:0099568: cytoplasmic region	GOCC:0099522: region of cytosol
GOCC:0030427: site of polarized growth	GOCC:0005911: cell-cell junction

Supplementary Table 10:

Functional annotations of total and differentially expressed phosphorylated proteins in neurons across sleep treatments.

Supplementary References

1. Diessler, S. *et al.* A systems genetics resource and analysis of sleep regulation in the mouse. *Plos Biol* 16, e2005750 (2018).

2. Scarpa, J. R. *et al.* Cross-species systems analysis identifies gene networks differentially altered by sleep loss and depression. *Sci Adv* 4, eaat1294 (2018).

3. Gerstner, J. R. *et al.* Removal of unwanted variation reveals novel patterns of gene expression linked to sleep homeostasis in murine cortex. *Bmc Genomics* 17, 727 (2016).
