# Supplementary material for: Single-cell transcriptomics and cell-specific proteomics reveals molecular signatures of sleep
Source: Commun Biol. 2022 Aug 19;5:846. doi: 10.1038/s42003-022-03800-3 (PMC9391396; doi:10.1038/s42003-022-03800-3)
Supplement: Supplementary file 3 — Description of Additional Supplementary Files [file 42003_2022_3800_MOESM3_ESM.docx]

**Description of Additional Supplementary Files**

**File name:** Supplementary Data 1.

**Description:** Summary of statistical tests performed (For Fig. 1b-d, Fig. 6b-f, and Supplementary Fig. 1a-e).

**File name:** Supplementary Data 2.

**Description:** Sleep phenotyping, scRNA-seq, and proteomics data analysis to identify the molecular signature of sleep. (The source data behind the graph of Fig. 1b, Fig. 3b-e, Fig. 4b-e, Fig. 5b-e, Fig. 6b-f, and Fig. 8b-c in the paper.)
